# Development and Validation of HPLC Method for the Simultaneous Determination of Five Food Additives and Caffeine in Soft Drinks

**DOI:** 10.1155/2016/2879406

**Published:** 2016-02-17

**Authors:** Bürge Aşçı, Şule Dinç Zor, Özlem Aksu Dönmez

**Affiliations:** Department of Chemistry, Faculty of Science and Arts, Yildiz Technical University, Davutpasa, 34220 Istanbul, Turkey

## Abstract

Box-Behnken design was applied to optimize high performance liquid chromatography (HPLC) conditions for the simultaneous determination of potassium sorbate, sodium benzoate, carmoisine, allura red, ponceau 4R, and caffeine in commercial soft drinks. The experimental variables chosen were pH (6.0–7.0), flow rate (1.0–1.4 mL/min), and mobile phase ratio (85–95% acetate buffer). Resolution values of all peak pairs were used as a response. Stationary phase was Inertsil OctaDecylSilane- (ODS-) 3V reverse phase column (250 × 4.6 mm, 5 *μ*m) dimensions. The detection was performed at 230 nm. Optimal values were found 6.0 pH, 1.0 mL/min flow rate, and 95% mobile phase ratio for the method which was validated by calculating the linearity (*r* ^2^ > 0.9962), accuracy (recoveries ≥ 95.75%), precision (intraday variation ≤ 1.923%, interday variation ≤ 1.950%), limits of detection (LODs), and limits of quantification (LOQs) parameters. LODs and LOQs for analytes were in the range of 0.10–0.19 *μ*g/mL and 0.33–0.63 *μ*g/mL, respectively. The proposed method was applied successfully for the simultaneous determination of the mixtures of five food additives and caffeine in soft drinks.

## 1. Introduction

Food additives are widely used in foodstuffs to prevent from spoilage and improve color, flavor, and texture of foods. However, these additives in foods may affect individuals who are sensitive with some type of allergy, asthma, and hay fever. Consequently, authorities have set threshold values for acceptable daily intake, varying from country to country. For instance, the list of authorised food additives and maximum permitted levels in European Union are laid down in the annexes of council directive [1, 2].

To ensure food safety from farm to fork, it is also essential to develop effective and reliable analytical methods for the monitoring of the additive levels in food [3]. Therefore, various analytical methods have been reported for the simultaneous determination synthetic food additives, such as thin layer chromatography [4], UV-visible spectrophotometry [5, 6], voltammetry [7, 8], differential pulse polarography [9], capillary electrophoresis [10], HPLC-DAD [11–14], HPLC-MS [15], and HPLC-MS-MS [16, 17]. Until now, although many analytical techniques have been developed for the determination of various food additives in foods, there is no report about simultaneous determination of this combination in food samples. Among these analytical methods, HPLC coupled with UV/Vis or diode array detectors (DADs) are the most commonly used methods due to their sensitivity, selectivity, and high resolution. So, development of effective chromatographic separation method involves judicious selection of experimental conditions that is suitable for the separation of interested components at an adequate resolution with reasonable run time. In this regard, experimental design is a useful tool to simplify the laborious work [18]. It not only is a timesaving method but also it has an ability to reveal possible interactions between variables [19, 20]. Hence, experimental designs have been increasingly used to determine the optimum conditions of chromatographic separation of some analytes in food, drug, and biological fluid samples with a minimum number of experiments for over the past decade [21–28].

In this paper, a new RP-HPLC method was developed, using experimental design, for simultaneous determination of five synthetic food additives in soft drinks, including three synthetic colorants (carmoisine, allura red, and ponceau 4R), two preservatives (potassium sorbate and sodium benzoate), and caffeine. For the optimization procedure, Box-Behnken design (BBD) was used to construct mathematical models that predict how changes input or controlled by variables (pH, flow rate, and mobile phase ratio) affected the resolution in defined experimental region. Further, the method validation has been carried out according to the International Conference on Harmonization guidelines. The optimized and validated method was successfully applied to some commercial soft drinks containing potassium sorbate, sodium benzoate, carmoisine, allura red, ponceau 4R, and caffeine.

## 2. Experimental

### 2.1. Apparatus

Chromatographic analyses were performed using a Shimadzu HPLC system (Kyoto, Japan) consisting of a model LC20 AT pump unit, SPD-20A UV-Vis detector, 7725 20 *μ*L sample injection, a computer, and an Inertsil OctaDecylSilane- (ODS-) 3V column (5 *μ*m, 250 mm × 4.6 mm; GL Sciences, Tokyo, Japan). The statistical analysis for the analytical responses and validation data was evaluated with Microsoft Excel 2000 software. The statistical software Statgraphics Centurion XV (StatPoint Inc., VA, USA) was used for the graph plotting and for estimating the responses of experimental variables.

### 2.2. Chemicals and Reagents

All chemicals and solvents were of analytical reagent grade and used without further purification. Milli-Q water was used to prepare the solutions and mobile phases (Millipore, Milford, MA, USA). Sodium acetate trihydrate, glacial acetic acid, and HPLC-grade acetonitrile were acquired from Merck (Darmstadt, Germany). Potassium sorbate (≥99.0% purity), sodium benzoate (≥99.0%, purity), carmoisine (≥98.0% purity), allura red (≥98.0% purity), ponceau 4R (≥99.0% purity), and caffeine (100.0% purity) were purchased from Sigma-Aldrich (St. Louis, Missouri, USA).

### 2.3. Preparation of Standard Solutions

Standard stock solutions of potassium sorbate, sodium benzoate, and caffeine were prepared at a concentration of 250 *μ*g/mL. Standard stock solutions of carmoisine, allura red, and ponceau 4R were prepared at a concentration of 100 *μ*g/mL. Fresh working solutions in the concentration range of 2–10 *μ*g/mL for carmoisine, allura red, and ponceau 4R and 5–25 *μ*g/mL for caffeine, potassium sorbate, and sodium benzoate were prepared by the dilution of the standard stock solutions in Milli-Q water.

### 2.4. Sample Preparation

Soft drink samples were purchased from local supermarkets in Istanbul, Turkey, and were degassed in an ultrasonic bath for 5 min. Then, 1 mL of the sample was transferred to a 10 mL volumetric flask and diluted to the volume with Milli-Q water. Prior to the analysis, both soft drink samples and standard solutions were filtered through 0.45 *μ*m Millipore filters and then injected into HPLC system.

### 2.5. Chromatographic Procedure

The optimum separation of all analytes was achieved with 0.025 M sodium acetate/acetic acid buffer, pH 6.0, acetonitrile gradient that follows 0–5 min, 95 : 75 (v/v); 5–10 min, 70 : 30 (v/v). The mobile phase flow rate was 1.0 mL/min and the injection volume was 20 *μ*L in all the chromatographic runs. The detection was made with a variable ultraviolet-visible detector fixed at 230 nm.

### 2.6. Optimization Procedure

A Box-Behnken design (BBD) using three variables at three levels (coded levels: −1, 0, and +1) was used for the optimization of simultaneous determination of potassium sorbate, sodium benzoate, carmoisine, allura red, ponceau 4R, and caffeine by HPLC. This design was selected due to the small number of experiments required. The variables and levels selected for optimization procedure were pH (*A*; 6.0, 6.5, and 7.0), flow rate (*B*; 1.0, 1.2, and 1.4), and mobile phase ratio (in terms of acetate buffer) (*C*; 85, 90, and 95) (Table 1). The proposed HPLC method analyzed the compounds in two steps as mentioned above. While the first step has an effect on the chromatographic separation, the second step has an effect on the run time of the method. Therefore, experimental variables of the first step of HPLC method were taken into account. 15 experimental runs were performed at random and overall resolution (*R*) was chosen as the response for the separation of the compounds [19]. Experimental design matrix used and the results obtained by BBD were listed in Table 2.

### 2.7. Validation Procedure

In-house validation of the method was performed according to International Conference on Harmonization guidelines (ICH Q2R1) [29]. Evaluated parameters are linearity of calibration curve, limit of detection (LOD), limit of quantification (LOQ), and precision, accuracy, and stability. The linearity of the HPLC method for the determination of five food additives and caffeine was evaluated in a concentration range of 2–10 *μ*g/mL for carmoisine, allura red, and ponceau 4R and 5–25 *μ*g/mL for potassium sorbate, sodium benzoate, and caffeine covering the normal range of concentrations obtained when analyzing soft drinks. Calibration equations were calculated by the least squares treatment of the peak area of the food additives and caffeine. The limit of detection (LOD) and limit of quantitation (LOQ) were calculated as LOD 3*xσ*/*S* and LOQ 10*xσ*/*S*, where *σ* is the standard deviation of intercept and *S* is the slope. In order to test the prediction performance of the proposed methods, intraday (three times in a day operation under the same conditions) and interday (four different days) studies were performed at three different concentrations (Level 1: 10 *μ*g/mL; Level 2: 15 *μ*g/mL; Level 3: 20 *μ*g/mL for potassium sorbate, sodium benzoate, and caffeine; Level 1: 4 *μ*g/mL; Level 2: 6 *μ*g/mL; Level 3: 8 *μ*g/mL for carmoisine, allura red, and ponceau 4R). Accuracy of the method was ascertained by a recovery study by adding a known amount of reference standards to the soft drink samples. Firstly, 0.5 mL of the soft drink sample was transferred to a 10 mL volumetric flask and the reference standards were added on it at three different concentration levels. Then, added samples were diluted to the volume with Milli-Q water, filtered, and analyzed.

## 3. Results and Discussion

### 3.1. Optimization of the HPLC Method

Chromatographic optimization requires selecting suitable criteria for the evaluation of the resultant chromatograms in order to choose the optimum conditions. BBD is an independent, rotatable, or nearly rotatable second-order design based on three-level incomplete factorial designs. It is more efficient compared to other response surface designs, such as central composite designs. It can also provide sufficient information to test the lack of fit, and therefore it is one of the best quadratic models for response surface method and has been widely used in analytical fields. Because of the nonlinearity of the model, a polynomial function to contain second-order model is postulated to describe the evolution phenomenon:

(1) $$\begin{array}{c} y_{i}=b_{0}+\overset{n}{\underset{i=1}{\sum }}b_{i}x_{i}+\overset{n}{\underset{i=1}{\sum }}b_{ii}x_{i}^{2}+\overset{n}{\underset{1\leq i\leq j}{\sum }}b_{ij}x_{i}x_{j}+\varepsilon_{i}, \end{array}$$

where *n* is the number of variables, *b*_0_ is the constant term, *b*_*i*_, *b*_*ii*_, and *b*_*ij*_ represent the coefficient of the first-order terms, quadratic terms, and interaction terms, respectively, and *ε*_*i*_ is a term that represents other sources of variability not accounted for the estimation, such as background noise [30].

**Figure 1:**
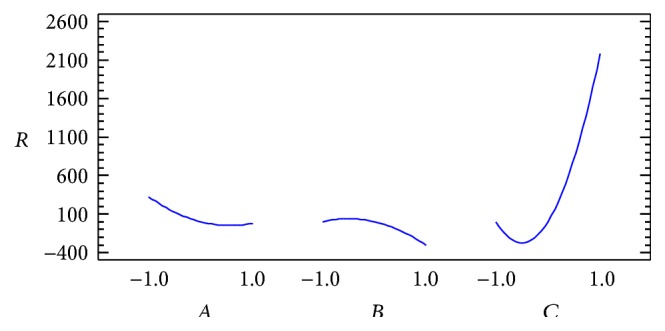
Analysis of the main variables in BBD.

The experimental results are shown in Table 2. The regression model for the response was tested through analysis of variance (ANOVA). From the results of ANOVA (Table 3), it can be deduced that linear contribution of mobile phase ratio (*C*) and quadratic contribution of mobile phase ratio (*CC*) influence the resolution significantly. Interactions of the individual variables in this study are not significant to resolution in the selected range. Fitted quadratic model equation is also presented in (2).  Figure 1 shows the analysis of individual variables of experimental design. From Figure 1, it can be seen how the value of the resolution may increase if we take higher mobile phase ratio (*C*). Also, we can infer that although pH (*A*) and flow rate (*B*) do not greatly influence the resolution better resolutions are obtained for low values of pH and flow rate

(2) R^=0.67−172.99A−153.92B+1089.10C+152.74A2−345.80AC−152.83B2−307.58BC+1088.49C2.

**Figure 2:**
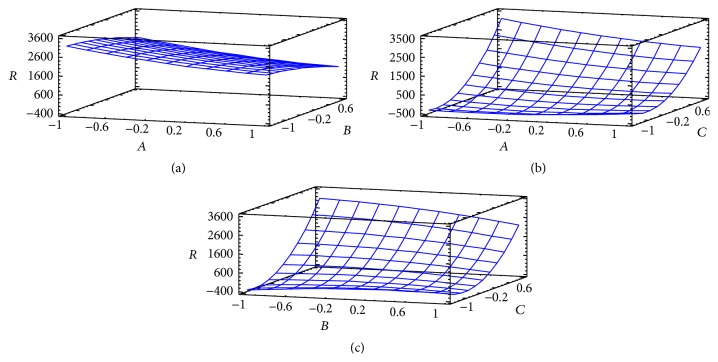
Response surface plots for BBD: (a) pH (*A*) versus flow rate (*B*) (mobile phase ratio: 95%); (b) pH (*A*) versus mobile phase ratio (*C*) (flow rate: 1.0 mL/min); (c) flow rate (*B*) versus mobile phase ratio (*C*) (pH: 6.0).

The regression models obtained were used to calculate the response surface for each variable separately.  Figure 2 illustrates the response surface plots for the resolutions. In particular, the effect of pH (*A*) and mobile phase ratio (*C*) on resolution is shown in Figure 2(b). This plot shows that the highest resolution is obtained at greater values of the mobile phase ratio. The relation between the effects of the other variables on the resolution is also plotted in Figures 2(a)–2(c).

**Figure 3:**
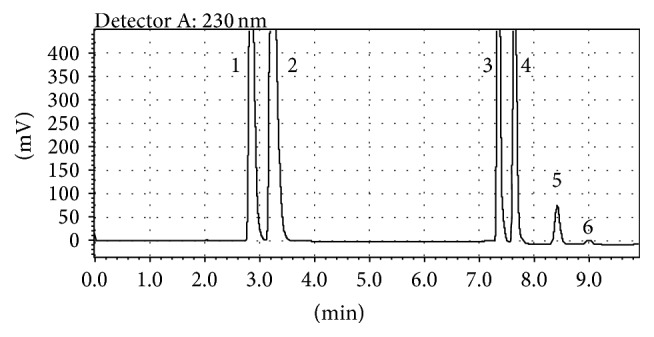
Chromatogram of synthetic standard mixture containing five food additives and caffeine recorded under optimized analysis conditions (1: sodium benzoate (15 *μ*g/mL), 2: potassium sorbate (15 *μ*g/mL), 3: caffeine (15 *μ*g/mL), 4: ponceau 4R (6 *μ*g/mL), 5: allura red (6 *μ*g/mL), and 6: carmoisine (6 *μ*g/mL)).

According to the results of the optimization procedure, the optimum variables corresponded to pH, 6.0; flow rate, 1.0 mL/min; mobile phase ratio, 95%. A typical chromatogram obtained under optimum conditions is shown in Figure 3.

### 3.2. Validation of the HPLC Method

The results of the linearity, LODs, and LOQs are summarized in Table 4. A good linear relationship is displayed between the corresponding peak areas and the concentrations of the compounds based on the correlation coefficients (*r* ^2^ > 0.9962). The LODs of the six compounds were in the range of 0.10–0.19 *μ*g/mL, and the LOQs of the six compounds were in the range of 0.33–0.63 *μ*g/mL. So, these values demonstrated that the proposed analytical method was sufficiently sensitive. A summary of intraday (the RSD of the recoveries of the nine samples) and interday precision (the RSD of the recoveries of the twelve samples) are listed in Table 5. The RSD values ranged from 0.310% to 1.950% for the HPLC method. These results show that the proposed method is precise for the simultaneous determination of these compounds. The recoveries of the six compounds to determine the accuracy of the method are summarized in Table 6. The proposed method resulted in satisfactory recoveries for all additives and caffeine, ranging from 97.67% to 105.56%. The recoveries demonstrated that the matrixes have negligible effect on the quantification of these compounds and the method is accurate within the desired range. Under refrigerated and room temperature conditions, all food additives and caffeine in mobile phase and water were stable for at least 1 month.

These results show that the proposed method is precise, accurate, and sensitive for the simultaneous determination of the six compounds and can be used for routine analysis of potassium sorbate, sodium benzoate, carmoisine, allura red, ponceau 4R, and caffeine in soft drinks.

### 3.3. Application of the Method

The proposed HPLC method was applied to the simultaneous determination of potassium sorbate, sodium benzoate, carmoisine, allura red, ponceau 4R, and caffeine in different soft drinks. Five replicates determination was made and the results are summarized in Table 7. The concentration of food additives in soft drinks ranged from 24.26 ± 0.47 *μ*g/mL to 254.13 ± 1.24 *μ*g/mL. The amounts of food additives and caffeine in all soft drink samples were below the limit value defined in the legislation on the food additives [1, 2].

## 4. Conclusion

An efficient, accurate, and reliable method for the simultaneous determination of five food additives and caffeine in soft drinks was developed using HPLC. Box-Behnken design was applied to the optimization of the chromatographic separation conditions and this design reduced to the number of experiments required. It can be concluded that a slight change in mobile phase ratio has a direct effect on the resolution. All the validation parameters were within the acceptance range. High percentage recovery data also shows that the proposed method is free from the interference. Consequently, this study will provide a sensitive and rapid method for the detection of potassium sorbate, sodium benzoate, carmoisine, allura red, ponceau 4R, and caffeine in soft drinks.

## Figures and Tables

**Table 1:** The experimental variables and levels of BBD.

Variable	Level		
	−1	0	+1
pH (*A*)	6.0	6.5	7.0
Flow rate (*B*) (mL min^−1^)	1.0	1.2	1.4
Mobile phase ratio (*C*) (%)	85	90	95

**Table 2:** Experimental design matrix and the responses for BBD.

Run	*A*	*B*	*C*	*R*
1	6.0	1.0	90	1.041
2	7.0	1.0	90	0.622
3	6.0	1.4	90	0.553
4	7.0	1.4	90	0.128
5	6.0	1.2	85	0.000
6	7.0	1.2	85	0.027
7	6.0	1.2	95	3175.373
8	7.0	1.2	95	1792.207
9	6.5	1.0	85	0.046
10	6.5	1.4	85	0.000
11	6.5	1.0	95	2487.823
12	6.5	1.4	95	1257.455
13	6.5	1.2	90	0.759
14	6.5	1.2	90	0.327
15	6.5	1.2	90	0.918

**Table 3:** ANOVA results for optimization by BBD.

Effect	SS	D.f.	MS	*F*-ratio	*P* value
*A*	239422	1	239422	1.95	0.2216
*B*	189543	1	189543	1.54	0.2694
*C*	9489050	1	9489050	77.20	0.0003^*∗*^
*AA*	86144	1	86144	0.70	0.4407
*AB*	0	1	0	0.00	1.0000
*AC*	478304	1	478304	3.89	0.1056
*BB*	86239	1	86239	0.70	0.4404
*BC*	378422	1	378422	3.08	0.1397
*CC*	4374670	1	4374670	35.59	0.0019^*∗*^
Total error	614539	5	122908		
Total (corr.)	15999393	14			

SS: sum of squares; MS: mean squares; *F*-ratio: MS/MS_error_; *P* value: probability level; D.f.: degree of freedom.

*R* ^2^ = 0.961, *R* ^2^ (adjusted for D.f.) = 0.892.

^*∗*^Significant factor at *α* = 0.05.

**Table 4:** The important parameters of the calibration equations for the proposed HPLC method for simultaneous determination of potassium sorbate (SOR), sodium benzoate (BEN), carmoisine (CAR), allura red (ALU), ponceau 4R (PON), and caffeine (CAF).

Compounds	Calibration range (*μ*g/mL)	Regression equation (*Y* = *aX* + *b*)	*S*_*a*_	*S*_*b*_	*r* ^2^	LOD (*μ*g/mL)	LOQ (*μ*g/mL)
SOR	5–25	*Y* = 7.8097 × 10^4^ *X* + 4.6484 × 10^4^	1.391 × 10^3^	2.3076 × 10^4^	0.9990	0.12	0.40
BEN	5–25	*Y* = 3.3313 × 10^4^ *X* + 1.2421 × 10^4^	7.52 × 10^2^	1.2469 × 10^4^	0.9985	0.10	0.33
CAR	2–10	*Y* = 4.3383 × 10^4^ *X* + 1.8834 × 10^4^	6.86 × 10^2^	4.555 × 10^3^	0.9992	0.16	0.53
ALU	2–10	*Y* = 5.5741 × 10^4^ *X* − 7.427 × 10^3^	9.22 × 10^2^	1.5292 × 10^4^	0.9992	0.11	0.35
PON	2–10	*Y* = 6.043 × 10^3^ *X* + 5.0389 × 10^4^	2.150 × 10^3^	1.4264 × 10^4^	0.9962	0.17	0.56
CAF	5–25	*Y* = 2.4885 × 10^4^ *X* + 4.6613 × 10^4^	4.81 × 10^2^	3.1891 × 10^4^	0.9989	0.19	0.63

*Y*: peak area; *X*: concentration (*μ*g/mL); *S*_*a*_: standard deviation of the slope; *S*_*b*_: standard deviation of the intercept.

**Table 5:** Validation of the simultaneous determination of food additives and caffeine by developed method potassium sorbate (SOR), sodium benzoate (BEN), carmoisine (CAR), allura red (ALU), ponceau 4R (PON), and caffeine (CAF).

Levels	SOR (*μ*g/mL)			BEN (*μ*g/mL)			CAR (*μ*g/mL)			ALU (*μ*g/mL)			PON (*μ*g/mL)			CAF (*μ*g/mL)		
	10.0	15.0	20.0	10.0	15.0	20.0	4.0	6.0	8.0	4.0	6.0	8.0	4.0	6.0	8.0	10.0	15.0	20.0
Intra-assay	9.97	15.22	19.76	9.70	15.37	20.18	4.05	6.17	7.92	3.90	6.02	7.86	3.79	5.72	7.75	9.96	15.15	20.28
	9.80	15.30	20.01	10.02	15.09	20.21	4.15	6.33	8.01	3.83	5.97	7.82	3.89	5.92	7.91	9.99	15.50	20.02
	9.91	15.31	19.86	10.03	14.79	20.38	4.18	6.20	8.11	3.87	6.06	7.70	3.92	5.87	8.04	10.03	15.16	20.20
Mean	9.89	15.28	19.88	9.92	15.08	20.26	4.13	6.23	8.01	3.87	6.02	7.79	3.87	5.84	7.91	9.99	15.27	20.16
RSD^a^%	0.869	0.321	0.634	1.895	1.923	0.533	1.646	1.364	1.186	0.904	0.747	1.060	1.757	1.781	1.643	0.350	0.310	0.689
Recovery%	98.90	101.87	99.40	99.20	100.53	101.30	103.25	103.83	100.13	96.75	100.33	97.83	96.75	97.33	98.88	99.90	101.80	100.80

Interassay	9.92	15.09	19.66	9.84	14.76	20.71	4.14	6.01	7.81	3.91	6.01	7.96	4.10	6.01	8.20	10.35	15.10	20.09
	9.84	15.23	19.59	9.85	14.95	20.74	4.18	6.06	8.14	3.85	5.84	7.90	4.08	6.18	8.26	10.01	15.32	20.43
	9.77	15.21	19.69	9.81	14.79	20.25	4.05	6.19	7.96	3.81	5.86	8.04	3.83	6.20	8.24	10.17	14.88	20.07
	9.68	15.40	19.89	10.02	14.82	20.11	4.19	6.22	7.98	3.76	6.08	7.82	4.01	5.98	7.99	10.03	15.16	20.20
Mean	9.80	15.23	19.71	9.88	14.83	20.45	4.14	6.12	7.97	3.83	5.95	7.93	4.03	6.09	8.14	10.14	15.11	20.20
RSD%	1.041	0.840	0.624	0.961	0.566	1.565	1.546	1.650	1.694	1.644	1.950	1.173	1.919	1.855	1.518	1.548	1.204	0.817
Recovery%	98.00	101.53	98.55	98.80	98.87	102.25	103.50	102.00	99.62	95.75	99.17	99.13	100.75	101.50	102.12	101.40	100.73	101.00

^a^Relative standard deviation, RSD (%), standard deviation/mean × 100.

**Table 6:** Results of accuracy studies (mean value ± standard deviation, *n* = 5).

Sample	Food additive	Sample concentration (*μ*g/mL)	Added (*μ*g/mL)	Found (*μ*g/mL)	Recovery (%)
Energy drink	Potassium sorbate	10.68	3	13.70 ± 0.048	100.67
			6	16.90 ± 0.057	103.67
			9	20.00 ± 0.065	103.56
	Caffeine	7.11	3	10.18 ± 0.045	102.33
			6	13.25 ± 0.054	102.33
			9	16.61 ± 0.042	105.56
	Allura red	3.26	1	4.31 ± 0.038	105.00
			2	5.34 ± 0.037	104.00
			3	6.39 ± 0.044	104.33

Pomegranate juice	Potassium sorbate	12.71	3	15.64 ± 0.059	97.67
			6	18.61 ± 0.103	98.33
			9	21.74 ± 0.166	100.34
	Sodium benzoate	7.24	3	10.34 ± 0.040	103.60
			6	13.30 ± 0.054	101.20
			9	16.45 ± 0.063	102.50
	Carmoisine	1.21	1	2.25 ± 0.012	104.00
			2	3.30 ± 0.017	104.50
			3	4.34 ± 0.022	104.40
	Ponceau 4R	4.08	1	5.11 ± 0.036	102.66
			2	6.16 ± 0.039	104.16
			3	7.15 ± 0.045	102.22

**Table 7:** Analysis of soft drinks (mean value ± standard deviation, *n* = 5).

Food additive	Energy drink (*μ*g/mL)	Pomegranate juice (*μ*g/mL)	Mandarin juice (*μ*g/mL)
Potassium sorbate	213.62 ± 0.34	254.13 ± 1.24	246.23 ± 1.76
Sodium benzoate	—	144.71 ± 2.38	148.67 ± 1.99
Caffeine	142.20 ± 1.17	—	—
Allura red	65.28 ± 0.59	—	—
Carmoisine	—	24.26 ± 0.47	—
Ponceau 4R	—	81.58 ± 1.51	—
